# Chlorido(4-methylpyridin-2-amine-κ*N* ^1^)(2-{[(4-methylpyridin-2-yl)imino-κ*N*]methyl}phenolato-κ*O*)copper(II)

**DOI:** 10.1107/S1600536812047198

**Published:** 2012-11-24

**Authors:** Bussa Bhagyaraju, P. Sambasiva Rao, Toka Swu

**Affiliations:** a Department of Chemistry, Pondicherry University, R.V. Nagar, Kalapet, Puducherry 605 014, India

## Abstract

In the title complex, [Cu(C_13_H_11_N_2_O)Cl(C_6_H_8_N_2_)], the Cu^II^ atom adopts a distorted tetra­hedral geometry being coordinated by the phenolic O atom and the azomethine N atom of the Schiff base ligand *N*-salicyl­idene 2-amino­pyridine, and by the 2-amino­pyridine N atom and a Cl atom. The pyridyl N atom of the Schiff base and the imino N atom of the 4-methyl-pyridine-2-yl­imino ligand are not involved in the coordination. There is an intra­molecular N—H⋯N hydrogen bond involving the pyridine N atom and the amino group of the 2-amino­pyridine ligand. In the crystal, mol­ecules are linked *via* N—H⋯Cl hydrogen bonds, forming chains propagating along [001].

## Related literature

For the preparation of similar compounds, see: Miao *et al.* (2009 ▶); Parashar *et al.* (1988 ▶); Castineiras *et al.* (1989 ▶). For the crystal structures of related compounds, see: Castineiras *et al.* (1989 ▶); Miao *et al.* (2009 ▶).
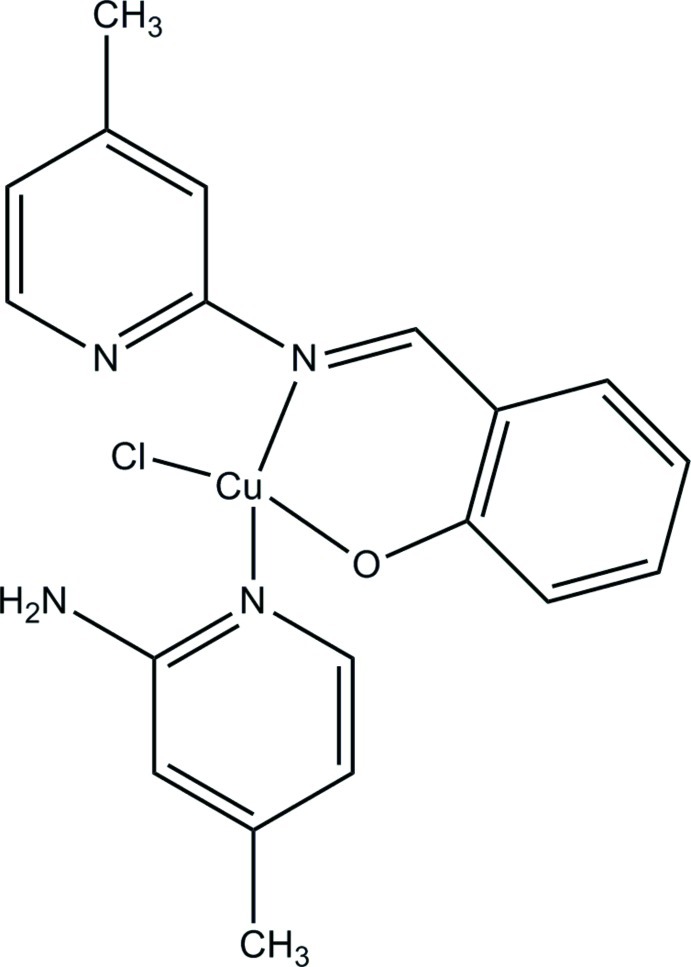


## Experimental

### 

#### Crystal data

[Cu(C_13_H_11_N_2_O)Cl(C_6_H_8_N_2_)
*M* *_r_* = 418.37
Monoclinic, 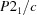

*a* = 17.443 (4) Å
*b* = 11.2197 (19) Å
*c* = 9.4435 (19) Å
β = 92.67 (2)°
*V* = 1846.1 (6) Å^3^
*Z* = 4
Mo *K*α radiation
μ = 1.34 mm^−1^
*T* = 300 K
0.40 × 0.40 × 0.06 mm

#### Data collection

Oxford Diffraction Xcalibur, Eos diffractometer
Absorption correction: multi-scan (*CrysAlis PRO*; Oxford Diffraction, 2010 ▶) *T*_min_ = 0.565, *T*_max_ = 1.000
7213 measured reflections
3339 independent reflections
2017 reflections with *I* > 2σ(*I*)
*R*_int_ = 0.063

#### Refinement

*R*[*F* ^2^ > 2σ(*F* ^2^)] = 0.055
*wR*(*F* ^2^) = 0.114
*S* = 0.94
3339 reflections
243 parameters
2 restraints
H atoms treated by a mixture of independent and constrained refinement
Δρ_max_ = 0.40 e Å^−3^
Δρ_min_ = −0.45 e Å^−3^

### 

Data collection: *CrysAlis PRO* (Oxford Diffraction, 2010 ▶); cell refinement: *CrysAlis PRO*; data reduction: *CrysAlis PRO*; program(s) used to solve structure: *SHELXS97* (Sheldrick, 2008 ▶); program(s) used to refine structure: *SHELXL97* (Sheldrick, 2008 ▶); molecular graphics: *OLEX2* (Dolomanov *et al.*, 2009 ▶) and *PLATON* (Spek, 2009 ▶); software used to prepare material for publication: *OLEX2*.

## Supplementary Material

Crystal structure: contains datablock(s) I, global. DOI: 10.1107/S1600536812047198/su2518sup1.cif

Structure factors: contains datablock(s) I. DOI: 10.1107/S1600536812047198/su2518Isup2.hkl

Additional supplementary materials:  crystallographic information; 3D view; checkCIF report

## Figures and Tables

**Table 1:** Hydrogen-bond geometry (Å, °)

*D*—H⋯*A*	*D*—H	H⋯*A*	*D*⋯*A*	*D*—H⋯*A*
N2—H2*NB*⋯N3	0.85 (4)	2.27 (4)	3.039 (6)	150 (5)
N2—H2*NA*⋯Cl1^i^	0.86 (4)	2.44 (4)	3.305 (5)	179 (7)

Symmetry code: (i) 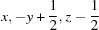.

**Fig. 1.**
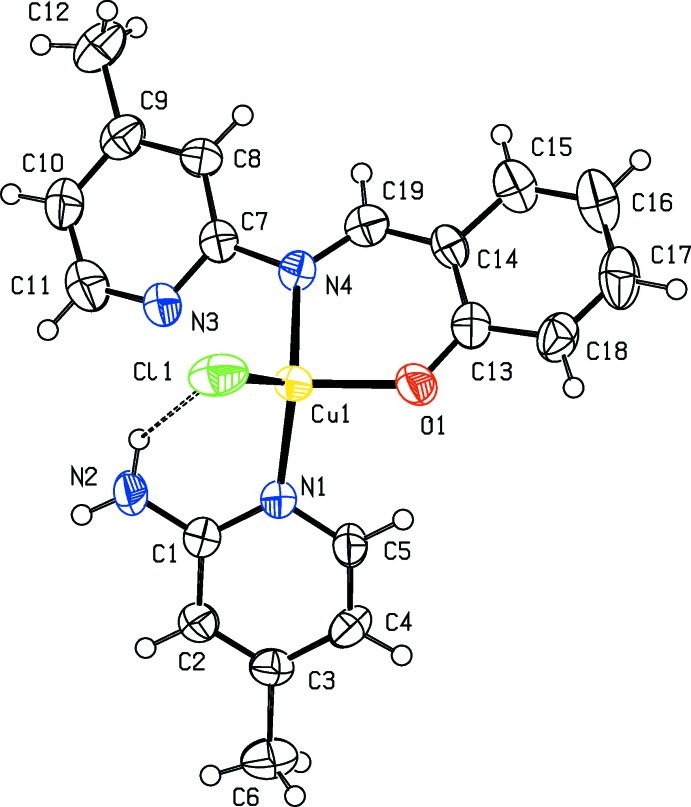
The molecular structure of title compound, showing the atom numbering. The displacement ellipsoids are drawn at the 50% probability level. The intramolecular N-H···Cl bond is shown as a dashed line (see Table 1 for details).

**Fig. 2.**
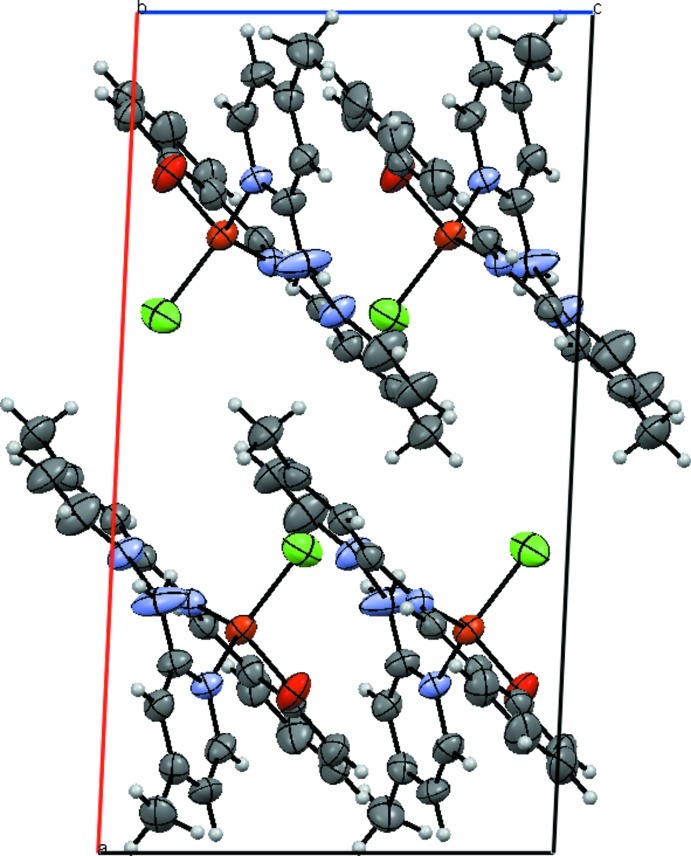
A view along b axis of the crystal packing of the title compound.

**Fig. 3.**
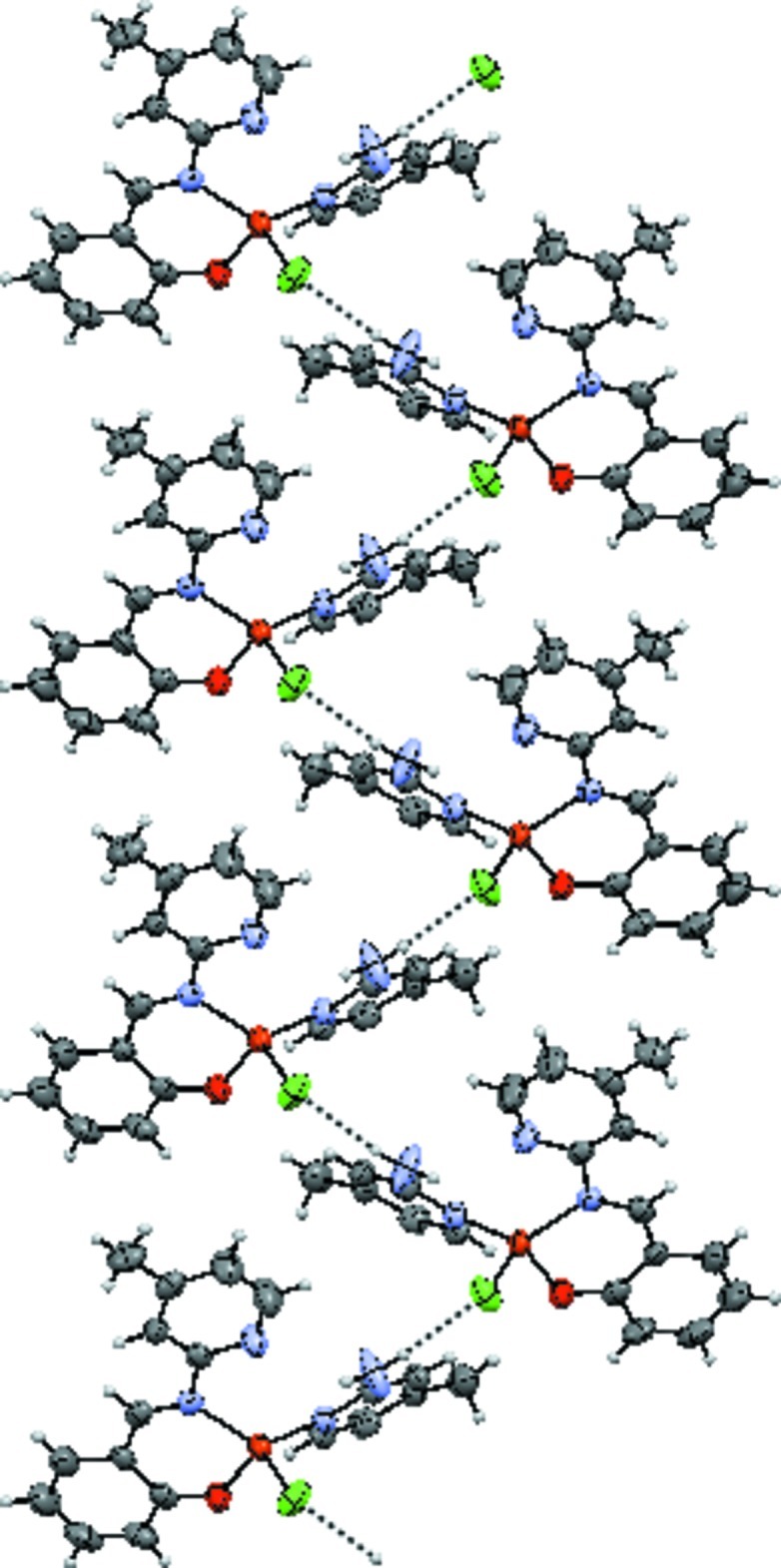
A view of the N-H···Cl hydrogen bonded chain structure propagating along the c axis direction (dashed line; see Table 1 for details).

## supplementary crystallographic information

### Comment

The Schiff base, *N*-salicylidene 2-aminopyridine, has been widely studied as a potential tridentate ligand. For example, the complex Bis{2-[(2-pyridyl)iminomethyl]-phenolato}copper(II), has been prepared by (Miao *et al.*, 2009), who reported that to a green solution of salicylaldehyde (0.19 mmol) and Cu(OAc)_2_.H_2_O (0.05 mmol) in ethanol they added slowly an enthanolic solution of 2-aminopyridine (0.22 mmol). The resulting mixture was allowed to stand and brown crystalline needles were obtained after 1 day. The same compound was prepared by an electrochemical method (Castineiras *et al.*, 1989) and by a solution method (Parashar *et al.*, 1988). We have used same procedure as (Miao *et al.*, 2009), but using a 1:1:1 molar ratio that produced the yellow crystals of the title compound, whose crystal structure we report on herein.

In the title complex, Fig. 1, the copper atom has a slightly distorted tetahedral geometry. It coordinates to the phenolic atom O1 and the azomethine atom N4 of the Schiff base liagnd *N*-salicylidene 2-aminopyridine, and to the 2-aminopyridine atom N1 and a chlorine atom, Cl1. The Cu—O1 and Cu—N4 bond lengths are similar to those reported in related structures (Miao *et al.*, 2009; Castineiras *et al.*, 1989). The structure of the molecule is stablized by an intramolecular N-H..Cl hydrogen bond (Table 1).

In the crystal, the intermolecular N-H···Cl hydrogen bond (Fig. 2 and Table 1) plays an important role in linking the molecules to form chains propagating along the c axis, as shown in Fig. 3.

### Experimental

A methanolic solution of 2-(((4-methyl-pyridine-2-yl)imino)methyl)phenol (0.01 moles) and 4-methylpyridin-2-amine (0.01 moles) was added slowly to a methanolic solution of copper chloride (0.01 moles). The resulting mixture was allowed to stand and yellow plate-like crystals were obtained after ca. 7 days.

### Refinement

The NH_2_ H-atoms were located in a difference Fourier map and refined with distances restraints: N-H = 0.86 (2) Å. The C-bound H atoms were positioned geometrically and refined using a riding model: C—H = 0.93 and 0.96 Å, for CH and CH_3_ H atoms, respectively; *U*_iso_ = k × U_eq_(N,C), where k = 1.5 for CH_3_ H atoms, and = 1.2 for other H atoms.

### Crystal data

**Table:**

[Cu(C_13_H_11_N_2_O)Cl(C_6_H_8_N_2_)]	*F*(000) = 860
*M**_r_* = 418.37	*D*_x_ = 1.505 Mg m^−^^3^
Monoclinic, *P*2_1_/*c*	Mo *K*α radiation, λ = 0.71070 Å
Hall symbol: -P 2ybc	Cell parameters from 2251 reflections
*a* = 17.443 (4) Å	θ = 2.8–29.4°
*b* = 11.2197 (19) Å	µ = 1.34 mm^−^^1^
*c* = 9.4435 (19) Å	*T* = 300 K
β = 92.67 (2)°	Plate, yellow
*V* = 1846.1 (6) Å^3^	0.4 × 0.4 × 0.06 mm
*Z* = 4	

### Data collection

**Table:**

Oxford Diffraction Xcalibur, Eos diffractometer	3339 independent reflections
Radiation source: Enhance (Mo) X-ray Source	2017 reflections with *I* > 2σ(*I*)
Graphite monochromator	*R*_int_ = 0.063
ω scans	θ_max_ = 25.3°, θ_min_ = 2.8°
Absorption correction: multi-scan (*CrysAlis PRO*; Oxford Diffraction, 2010)	*h* = −14→20
*T*_min_ = 0.565, *T*_max_ = 1.000	*k* = −13→10
7213 measured reflections	*l* = −11→11

### Refinement

**Table:**

Refinement on *F*^2^	Primary atom site location: structure-invariant direct methods
Least-squares matrix: full	Secondary atom site location: difference Fourier map
*R*[*F*^2^ > 2σ(*F*^2^)] = 0.055	Hydrogen site location: inferred from neighbouring sites
*wR*(*F*^2^) = 0.114	H atoms treated by a mixture of independent and constrained refinement
*S* = 0.94	*w* = 1/[σ^2^(*F*_o_^2^) + (0.0352*P*)^2^] where *P* = (*F*_o_^2^ + 2*F*_c_^2^)/3
3339 reflections	(Δ/σ)_max_ = 0.001
243 parameters	Δρ_max_ = 0.40 e Å^−^^3^
2 restraints	Δρ_min_ = −0.45 e Å^−^^3^

### Special details

**Table:**

Geometry. Bond distances, angles etc. have been calculated using the rounded fractional coordinates. All su's are estimated from the variances of the (full) variance-covariance matrix. The cell esds are taken into account in the estimation of distances, angles and torsion angles
Refinement. Refinement of F^2^ against ALL reflections. The weighted R-factor wR and goodness of fit S are based on F^2^, conventional R-factors R are based on F, with F set to zero for negative F^2^. The threshold expression of F^2^ > 2sigma(F^2^) is used only for calculating R-factors(gt) etc. and is not relevant to the choice of reflections for refinement. R-factors based on F^2^ are statistically about twice as large as those based on F, and R- factors based on ALL data will be even larger.

### Fractional atomic coordinates and isotropic or equivalent isotropic displacement parameters (Å^2^)

**Table:**

	*x*	*y*	*z*	*U*_iso_*/*U*_eq_	
Cu1	0.23453 (3)	0.51797 (4)	0.79066 (6)	0.0389 (2)	
Cl1	0.13907 (8)	0.44704 (11)	0.91654 (15)	0.0642 (5)	
O1	0.30823 (19)	0.6041 (3)	0.9125 (3)	0.0528 (13)	
N1	0.3004 (2)	0.3846 (3)	0.7218 (4)	0.0385 (12)	
N2	0.2006 (3)	0.2818 (4)	0.6107 (6)	0.079 (2)	
N3	0.1439 (2)	0.5299 (3)	0.5301 (5)	0.0526 (16)	
N4	0.2022 (2)	0.6618 (3)	0.6805 (4)	0.0367 (12)	
C1	0.2746 (3)	0.2872 (4)	0.6490 (5)	0.0436 (17)	
C2	0.3246 (3)	0.1932 (4)	0.6186 (5)	0.0432 (17)	
C3	0.3996 (3)	0.1993 (4)	0.6589 (5)	0.0420 (17)	
C4	0.4263 (3)	0.3013 (4)	0.7295 (5)	0.0476 (17)	
C5	0.3758 (3)	0.3887 (4)	0.7583 (5)	0.0433 (17)	
C6	0.4539 (3)	0.0991 (4)	0.6281 (5)	0.061 (2)	
C7	0.1478 (3)	0.6438 (4)	0.5666 (5)	0.0389 (17)	
C8	0.1030 (3)	0.7309 (4)	0.5036 (5)	0.0424 (17)	
C9	0.0524 (3)	0.7024 (4)	0.3933 (5)	0.0476 (17)	
C10	0.0505 (3)	0.5841 (4)	0.3494 (6)	0.067 (2)	
C11	0.0968 (4)	0.5026 (5)	0.4217 (7)	0.077 (3)	
C12	0.0006 (3)	0.7932 (5)	0.3246 (6)	0.066 (2)	
C13	0.3222 (3)	0.7167 (4)	0.9098 (5)	0.0421 (17)	
C14	0.2837 (3)	0.7994 (4)	0.8159 (5)	0.0406 (17)	
C15	0.3030 (3)	0.9213 (4)	0.8254 (5)	0.0577 (19)	
C16	0.3574 (4)	0.9616 (5)	0.9200 (6)	0.072 (3)	
C17	0.3958 (3)	0.8815 (5)	1.0102 (6)	0.066 (2)	
C18	0.3778 (3)	0.7637 (4)	1.0062 (5)	0.053 (2)	
C19	0.2287 (3)	0.7673 (4)	0.7090 (5)	0.0428 (17)	
H2NA	0.184 (3)	0.223 (3)	0.559 (5)	0.0950*	
H2	0.30570	0.12630	0.57040	0.0520*	
H2NB	0.177 (3)	0.348 (3)	0.618 (6)	0.0950*	
H4	0.47800	0.30930	0.75630	0.0570*	
H5	0.39440	0.45580	0.80650	0.0520*	
H6A	0.42650	0.03750	0.57640	0.0920*	
H6B	0.47520	0.06720	0.71560	0.0920*	
H6C	0.49450	0.12870	0.57260	0.0920*	
H8	0.10680	0.80920	0.53560	0.0510*	
H10	0.01860	0.56040	0.27290	0.0800*	
H11	0.09480	0.42360	0.39210	0.0930*	
H12A	−0.02900	0.83080	0.39510	0.0990*	
H12B	−0.03320	0.75510	0.25550	0.0990*	
H12C	0.03080	0.85210	0.27880	0.0990*	
H15	0.27770	0.97530	0.76500	0.0700*	
H16	0.36900	1.04250	0.92470	0.0850*	
H17	0.43420	0.90870	1.07380	0.0780*	
H18	0.40330	0.71230	1.06970	0.0640*	
H19	0.20910	0.82900	0.65230	0.0520*	

### Atomic displacement parameters (Å^2^)

**Table:**

	*U*^11^	*U*^22^	*U*^33^	*U*^12^	*U*^13^	*U*^23^
Cu1	0.0416 (4)	0.0315 (3)	0.0429 (4)	0.0040 (3)	−0.0052 (3)	−0.0010 (3)
Cl1	0.0576 (10)	0.0592 (8)	0.0768 (10)	0.0139 (7)	0.0154 (8)	0.0244 (8)
O1	0.067 (3)	0.0363 (18)	0.053 (2)	0.0006 (16)	−0.0208 (19)	−0.0021 (17)
N1	0.035 (2)	0.034 (2)	0.046 (2)	−0.0017 (18)	−0.004 (2)	−0.0007 (19)
N2	0.043 (3)	0.052 (3)	0.139 (5)	0.006 (2)	−0.026 (3)	−0.049 (3)
N3	0.053 (3)	0.041 (2)	0.062 (3)	−0.003 (2)	−0.016 (2)	−0.006 (2)
N4	0.034 (2)	0.040 (2)	0.036 (2)	−0.0026 (18)	0.0004 (19)	0.0008 (19)
C1	0.037 (3)	0.040 (3)	0.053 (3)	−0.001 (2)	−0.005 (3)	−0.003 (3)
C2	0.045 (3)	0.040 (3)	0.044 (3)	0.003 (2)	−0.003 (3)	−0.005 (2)
C3	0.046 (3)	0.039 (3)	0.041 (3)	0.008 (2)	0.003 (3)	−0.001 (2)
C4	0.033 (3)	0.060 (3)	0.049 (3)	0.008 (3)	−0.006 (3)	0.000 (3)
C5	0.042 (3)	0.041 (3)	0.046 (3)	−0.008 (2)	−0.009 (3)	−0.003 (2)
C6	0.060 (4)	0.061 (3)	0.063 (4)	0.021 (3)	0.008 (3)	0.001 (3)
C7	0.038 (3)	0.042 (3)	0.037 (3)	−0.004 (2)	0.006 (2)	−0.004 (2)
C8	0.045 (3)	0.037 (3)	0.045 (3)	0.001 (2)	−0.001 (3)	0.005 (2)
C9	0.037 (3)	0.055 (3)	0.051 (3)	−0.003 (3)	0.004 (3)	0.015 (3)
C10	0.062 (4)	0.059 (4)	0.077 (4)	−0.010 (3)	−0.026 (3)	−0.006 (3)
C11	0.084 (5)	0.053 (3)	0.092 (5)	−0.005 (3)	−0.027 (4)	−0.015 (3)
C12	0.053 (4)	0.086 (4)	0.058 (4)	0.008 (3)	−0.011 (3)	0.013 (3)
C13	0.038 (3)	0.051 (3)	0.038 (3)	−0.005 (2)	0.008 (3)	−0.009 (3)
C14	0.048 (3)	0.037 (3)	0.037 (3)	−0.006 (2)	0.004 (3)	−0.009 (2)
C15	0.069 (4)	0.051 (3)	0.053 (3)	−0.014 (3)	0.002 (3)	−0.003 (3)
C16	0.084 (5)	0.068 (4)	0.062 (4)	−0.030 (4)	−0.001 (4)	−0.011 (3)
C17	0.065 (4)	0.083 (4)	0.049 (4)	−0.019 (3)	0.001 (3)	−0.026 (3)
C18	0.049 (4)	0.067 (4)	0.043 (3)	0.002 (3)	−0.008 (3)	−0.014 (3)
C19	0.041 (3)	0.046 (3)	0.042 (3)	0.001 (2)	0.007 (3)	0.005 (3)

### Geometric parameters (Å, º)

**Table:**

Cu1—Cl1	2.2368 (16)	C13—C18	1.402 (7)
Cu1—O1	1.942 (3)	C13—C14	1.429 (7)
Cu1—N1	2.013 (4)	C14—C19	1.407 (7)
Cu1—N3	2.865 (5)	C14—C15	1.410 (6)
Cu1—N4	1.987 (4)	C15—C16	1.351 (8)
O1—C13	1.287 (6)	C16—C17	1.389 (8)
N1—C1	1.357 (6)	C17—C18	1.359 (7)
N1—C5	1.345 (6)	C2—H2	0.9300
N2—C1	1.326 (7)	C4—H4	0.9300
N3—C7	1.325 (6)	C5—H5	0.9300
N3—C11	1.319 (8)	C6—H6A	0.9600
N4—C7	1.415 (6)	C6—H6B	0.9600
N4—C19	1.295 (6)	C6—H6C	0.9600
N2—H2NB	0.85 (4)	C8—H8	0.9300
N2—H2NA	0.86 (4)	C10—H10	0.9300
C1—C2	1.407 (7)	C11—H11	0.9300
C2—C3	1.347 (7)	C12—H12A	0.9600
C3—C6	1.507 (7)	C12—H12B	0.9600
C3—C4	1.394 (7)	C12—H12C	0.9600
C4—C5	1.354 (7)	C15—H15	0.9300
C7—C8	1.370 (7)	C16—H16	0.9300
C8—C9	1.371 (7)	C17—H17	0.9300
C9—C10	1.391 (6)	C18—H18	0.9300
C9—C12	1.490 (7)	C19—H19	0.9300
C10—C11	1.380 (8)		
			
Cl1—Cu1—O1	110.53 (10)	C14—C13—C18	116.7 (4)
Cl1—Cu1—N1	110.94 (11)	C13—C14—C15	119.1 (4)
Cl1—Cu1—N3	94.50 (9)	C13—C14—C19	124.3 (4)
Cl1—Cu1—N4	111.53 (11)	C15—C14—C19	116.5 (4)
O1—Cu1—N1	100.91 (14)	C14—C15—C16	121.7 (5)
O1—Cu1—N3	144.00 (12)	C15—C16—C17	119.5 (5)
O1—Cu1—N4	94.01 (14)	C16—C17—C18	120.6 (5)
N1—Cu1—N3	93.31 (13)	C13—C18—C17	122.4 (5)
N1—Cu1—N4	125.93 (15)	N4—C19—C14	127.5 (4)
N3—Cu1—N4	51.74 (13)	C1—C2—H2	120.00
Cu1—O1—C13	126.7 (3)	C3—C2—H2	120.00
Cu1—N1—C1	125.6 (3)	C3—C4—H4	121.00
Cu1—N1—C5	117.3 (3)	C5—C4—H4	120.00
C1—N1—C5	117.1 (4)	N1—C5—H5	118.00
Cu1—N3—C7	78.6 (3)	C4—C5—H5	118.00
Cu1—N3—C11	162.7 (3)	C3—C6—H6A	109.00
C7—N3—C11	116.7 (4)	C3—C6—H6B	109.00
Cu1—N4—C7	116.5 (3)	C3—C6—H6C	109.00
Cu1—N4—C19	122.9 (3)	H6A—C6—H6B	109.00
C7—N4—C19	120.6 (4)	H6A—C6—H6C	109.00
H2NA—N2—H2NB	124 (5)	H6B—C6—H6C	109.00
C1—N2—H2NB	114 (3)	C7—C8—H8	120.00
C1—N2—H2NA	119 (3)	C9—C8—H8	120.00
N2—C1—C2	121.0 (4)	C9—C10—H10	121.00
N1—C1—N2	118.1 (4)	C11—C10—H10	121.00
N1—C1—C2	120.9 (5)	N3—C11—H11	118.00
C1—C2—C3	120.5 (4)	C10—C11—H11	118.00
C2—C3—C4	118.4 (4)	C9—C12—H12A	109.00
C2—C3—C6	121.2 (4)	C9—C12—H12B	109.00
C4—C3—C6	120.4 (5)	C9—C12—H12C	109.00
C3—C4—C5	119.0 (5)	H12A—C12—H12B	110.00
N1—C5—C4	124.2 (4)	H12A—C12—H12C	110.00
N3—C7—C8	123.6 (5)	H12B—C12—H12C	109.00
N3—C7—N4	111.1 (4)	C14—C15—H15	119.00
N4—C7—C8	125.2 (4)	C16—C15—H15	119.00
C7—C8—C9	119.8 (4)	C15—C16—H16	120.00
C8—C9—C10	117.0 (4)	C17—C16—H16	120.00
C10—C9—C12	121.2 (5)	C16—C17—H17	120.00
C8—C9—C12	121.8 (4)	C18—C17—H17	120.00
C9—C10—C11	118.7 (5)	C13—C18—H18	119.00
N3—C11—C10	124.0 (5)	C17—C18—H18	119.00
O1—C13—C14	124.4 (4)	N4—C19—H19	116.00
O1—C13—C18	118.8 (4)	C14—C19—H19	116.00
			
Cl1—Cu1—O1—C13	−113.3 (4)	C11—N3—C7—C8	3.8 (8)
N1—Cu1—O1—C13	129.3 (4)	C7—N3—C11—C10	−2.4 (9)
N3—Cu1—O1—C13	17.9 (5)	Cu1—N4—C7—N3	−17.2 (5)
N4—Cu1—O1—C13	1.5 (4)	Cu1—N4—C7—C8	161.1 (4)
Cl1—Cu1—N1—C1	52.5 (4)	C19—N4—C7—N3	162.7 (4)
Cl1—Cu1—N1—C5	−123.3 (3)	C19—N4—C7—C8	−19.0 (7)
O1—Cu1—N1—C1	169.6 (4)	Cu1—N4—C19—C14	1.3 (7)
O1—Cu1—N1—C5	−6.1 (3)	C7—N4—C19—C14	−178.6 (5)
N3—Cu1—N1—C1	−43.6 (4)	N1—C1—C2—C3	−1.3 (7)
N3—Cu1—N1—C5	140.6 (3)	N2—C1—C2—C3	−179.5 (5)
N4—Cu1—N1—C1	−87.2 (4)	C1—C2—C3—C4	−0.8 (7)
N4—Cu1—N1—C5	97.0 (4)	C1—C2—C3—C6	179.6 (4)
Cl1—Cu1—N3—C7	104.9 (3)	C2—C3—C4—C5	1.8 (7)
O1—Cu1—N3—C7	−30.1 (4)	C6—C3—C4—C5	−178.6 (4)
N1—Cu1—N3—C7	−143.8 (3)	C3—C4—C5—N1	−0.7 (7)
N4—Cu1—N3—C7	−9.1 (3)	N3—C7—C8—C9	−2.2 (8)
Cl1—Cu1—N4—C7	−68.9 (3)	N4—C7—C8—C9	179.8 (5)
Cl1—Cu1—N4—C19	111.2 (4)	C7—C8—C9—C10	−1.1 (7)
O1—Cu1—N4—C7	177.2 (3)	C7—C8—C9—C12	177.9 (5)
O1—Cu1—N4—C19	−2.7 (4)	C8—C9—C10—C11	2.4 (8)
N1—Cu1—N4—C7	70.6 (4)	C12—C9—C10—C11	−176.6 (5)
N1—Cu1—N4—C19	−109.3 (4)	C9—C10—C11—N3	−0.7 (9)
N3—Cu1—N4—C7	9.4 (3)	O1—C13—C14—C15	178.6 (5)
N3—Cu1—N4—C19	−170.5 (4)	O1—C13—C14—C19	−3.8 (8)
Cu1—O1—C13—C14	1.3 (7)	C18—C13—C14—C15	−0.3 (7)
Cu1—O1—C13—C18	−179.9 (3)	C18—C13—C14—C19	177.3 (5)
Cu1—N1—C1—N2	4.9 (6)	O1—C13—C18—C17	−179.9 (5)
Cu1—N1—C1—C2	−173.4 (3)	C14—C13—C18—C17	−1.0 (8)
C5—N1—C1—N2	−179.4 (5)	C13—C14—C15—C16	0.6 (8)
C5—N1—C1—C2	2.4 (6)	C19—C14—C15—C16	−177.2 (5)
Cu1—N1—C5—C4	174.7 (4)	C13—C14—C19—N4	2.4 (9)
C1—N1—C5—C4	−1.4 (7)	C15—C14—C19—N4	−179.9 (5)
Cu1—N3—C7—N4	10.8 (3)	C14—C15—C16—C17	0.3 (9)
Cu1—N3—C7—C8	−167.5 (5)	C15—C16—C17—C18	−1.6 (9)
C11—N3—C7—N4	−177.9 (5)	C16—C17—C18—C13	2.0 (8)

### Hydrogen-bond geometry (Å, º)

**Table:**

*D*—H···*A*	*D*—H	H···*A*	*D*···*A*	*D*—H···*A*
N2—H2*NB*···N3	0.85 (4)	2.27 (4)	3.039 (6)	150 (5)
N2—H2*NA*···Cl1^i^	0.86 (4)	2.44 (4)	3.305 (5)	179 (7)

Symmetry code: (i) *x*, −*y*+1/2, *z*−1/2.
